# Household perception and infestation dynamics of bedbugs among residential communities and its potential distribution in Africa

**DOI:** 10.1038/s41598-022-24339-7

**Published:** 2022-11-18

**Authors:** Dennis M. Mbuta, Fathiya M. Khamis, Bonoukpoè M. Sokame, Florence Ng’ong’a, Komivi S. Akutse

**Affiliations:** 1 International Centre of Insect Physiology and Ecology, P.O. Box 30772-00100, Nairobi, Kenya; 2 Jomo Kenyatta University of Agriculture and Technology, P.O. Box 62000-00200, Nairobi, Kenya

**Keywords:** Biological techniques, Ecology, Zoology, Ecology, Environmental sciences, Environmental social sciences, Mathematics and computing

## Abstract

Bedbugs have experienced an extraordinary upsurge in the recent past across the world. This cross-sectional study aimed to explore the community perception of the pest outbreaks, the population dynamics, and dispersal patterns under different habitat systems. A survey was conducted within communities in nine counties in Kenya, where geographical coordinates of the sites of bedbug presence were recorded and maximum entropy distribution modelling (MaxEnt) was used to map and predict the potentially suitable habitat, while system thinking and system dynamics approach with Vensim PLE 8.0.9 software was applied to implement bedbug infestation dynamics. Our results indicated that majority of the respondents had ample knowledge on bedbugs and were concerned about the physico-psychologic and socio-economic health effects. Spatial distribution analysis showed regions in Kenya with optimal to suitable for bedbug occurrence in the whole country, and similar results were found at continental level across Africa. Furthermore, infestation dynamics results showed a rapid mobility of bedbug from one house to another. In terms of management strategies, the models showed that the combination of chemical with other control methods was considerably much more effective compared to the use of chemical approach only, appointing integrated pest management strategy as a better intervention approach in controlling the pest.

## Introduction

Bedbugs *Cimex* spp. (Hemipteria: Cimicidae) are ectoparasites and obligate hematophagous arthropods^1^. They are characterized by their ovoid, wingless, red-brown, and dorso-ventrally flattened bodies with the ability to grow up to 7 mm in length and a lifespan of four months up to 1 year^2^. The pests are normally cryptic, photophobic, and thigmotactic with a typically buggy and sickly sweet odor^1^. In addition to the bulky smell, the presence of faecal spots, empty egg cases, eggs, nymphs, shed exoskeletons, and dead or live bedbugs are quick signs of an emerging infestation^3^. Among the species of family Cimicidae, the common bedbug (*Cimex lectularius*) and the tropical bedbug (*Cimex hemipterus*) have been identified to entirely depend on human bloodmeal^1^. The two are very similar and prevalent in temperate, tropical and subtropical regions (*C. lectularius* 28°–29°, *C. hemipterus* 32–33 °C)^4^; but both species can be found beyond their normal ranges^5^. Bedbugs usually hide in harbourages such as cracks and crevices of wooded furniture, beds, wall voids, under-fitted carpets, behind pictures, skirting boards and headboards, seams on mattresses, in bed-frames, inside electrical fittings, and in curtains^6^. However, this nuisance pest can infest almost everywhere people frequently reside. This societal pest is usually attracted to their hosts by body heat, exhaled carbon dioxide, and other compounds emitted by the skin. Bedbugs also feed on other warm-blooded animals, such as pets, though humans are the preferred hosts^1^.

Bedbugs are reported to have started associating with human beings at least 10,000 years ago^7^ and their infestations come with unbearable consequences. Among them include bites on the arms and legs. However, these bites can occur on any exposed part of the skin; they normally appear as erythematous papules with a central punctum and 2–5 mm pruritic in either linear, sequential, or clustered arrangements in a “breakfast, lunch and dinner pattern”^8^. For long, bedbugs have been hypothesized as potential disease vectors for nematodes, protozoans, bacteria, and viruses^1^. Additionally, past studies have raised suspicion of bedbugs serving as vectors for almost 45 human infectious diseases but with no casual evidence as at present^9^. Also, they have been proved unable to transmit non-arthropod-borne viral pathogens such as HIV, hepatitis B, C and E viruses^1^, though with an ability to retain HIV for approximately eight days and HBV for at least 7.5 weeks^10^. It is worrisome that other members of the family Cimicidae including, swallow bugs (*Oeciacus vicarious* Horvath) are known as biological vectors for arboviruses i.e. Stone Lake Virus^11^, Fort Morgan virus^12^, and Buggy Creek virus^13^. Furthermore, the arbovirus Kaeng Khoi is transmittable to bats by the bat bugs *Stricticimex parvus* Ueshima and *Cimex insuetus* Ueshima^14^*.* If other Cimicidae families have the ability to transmit pathogens, why not bedbugs? This question warranted other additional studies to trace the various possible pathways. Although they may appear painless, the bites might cause severe reactions ranging from asymptomatic to itchy, swollen, and blistered, often causing secondary bacterial infections^15^ such as lymphangitis, ecthyma, impetigo, and cellulitis^4^. Nevertheless, psychological effects like paranoia, insomnia, stress, anger, anxiety, emotional distress, embarrassment^1,16^, delusions, mood changes, panic, social withdrawal, post-traumatic stress disorder (PTSD) and some reports of suicidality have been documented^17,18^. The pest has been reported to take a large blood meal of about 13.2 ml with a possibility of resulting in anemia during high infestation instances^19^. The infested hosts usually have different reactions to the bug's saliva, where approximately 70% have been linked to related allergic effects such as urticaria, itchmen erythematous, and popular lesions^20^. In some instances, the infestations have entirely compromised people’s lives. This has resulted in mental health due to agitation, paranoia, emotional distress, insomnia, depression, and even suicidal thoughts^16^. There have been high levels of stigma and social isolation among the victims since the pest is associated with poor housekeeping and hygiene. A long-term psychological trauma out of the bites can lead to a delusionary state to the patient. The nuisance nature of this indoor pest has since been compared to rats which have been classified as more socially acceptable than the bedbugs^1^.

Bedbug infestation affects all socioeconomic backgrounds, which comes along with financial distress. This has led to serious economic consequences as a result of being a socioeconomic burden in society. The disinfestation process has been pointed out as an expensive exercise since it involves constant inspection, quarantine of infested areas, treatments, disposal, and replacement of infested household items as well as other furnishings^21^. Past studies have reported serious economic consequences, especially in the hospitality and tourism sectors^22^. In 2010, a survey in the United States reported that 800–1200 USD were used for disinfestation for every infested area^7^, while in Australia, an estimate of AUS$100 million was used in 2006^23^. Furthermore, it was approximated that a cost amounting to 2500–3000 USD would be spent in disinfection and replacement of infested belongings using a standard insecticide^24^. Also, the government of Ghana is reported to have spent several millions of Cedis (1 Cedis = USD$0.17) in the disinfestation process across the country^25^. The process of developing and marketing a new active insecticidal agent is costly with an estimated cost of over US$ 180 million^1^.

The fight against eradicating bedbugs’ infestation has never been prioritized in many of the affected countries. The less developed countries, mainly characterized by poor health infrastructure have higher entomological priorities with potentially fatal endemic vector-borne diseases such as dengue and malaria to combat. Therefore, bedbugs being a public health pest and not a disease vector, end up mainly being ignored^26^. Furthermore, despite the availability of powerful tools to shed light on the biology, ecology, and management of the pest, the infestation dynamics of those primarily commensal within human dwelling remains largely unaddressed; especially in Kenya where even the diversity of the pest species is still unknown, but the outbreaks of the pest have been reported in households, universities, transport and hospitality industries. The present study has objectives to (1) determine the prevalence and knowledge of bedbug infestations among resident communities, assess the current management approaches and its socio-economic impacts; (2) study the dynamics of bedbug infestation in residential communities and simulate the effect of different control strategies; and (3) predict the potential geographical distribution of bedbug in Africa and analyze the suitable environment factors affecting the distribution of the pest.

## Results

### Bedbug Genus identification

**Figure 1:**
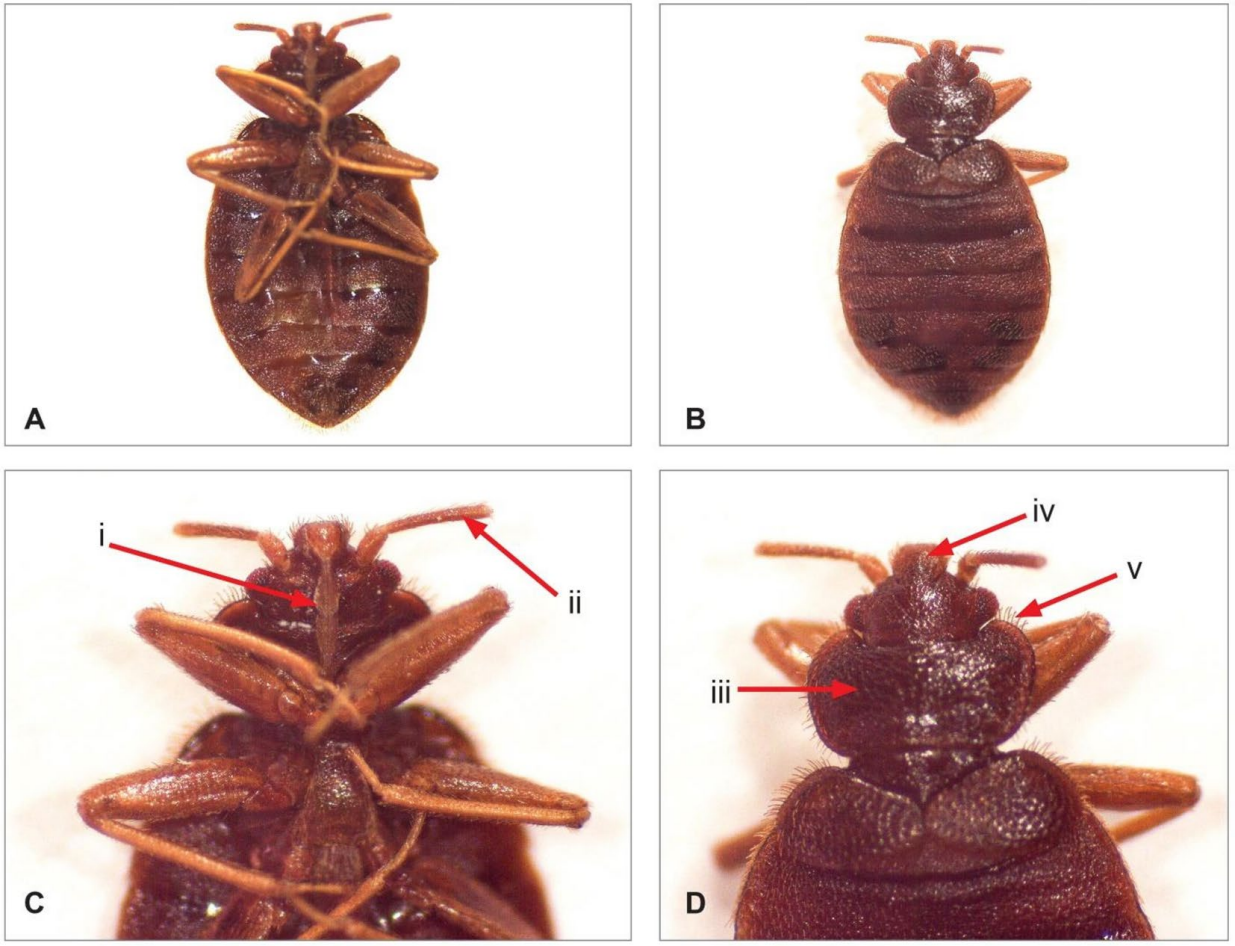
Morphological features of bedbug: (**A**) ventral side, (**B**) dorsal side, (**C**) ventral head section; (i) proboscis; (ii) antenna, (**D**) dorsal thoracic section; (iii) pronotum; (iv) Labrum; (v) fine hairs.

Our samples bore the defined characteristics of *Cimex* genus of bedbug as detailed in the monograph. The adult bedbugs were wingless, dorsoventrally flattened, and brown in appearance (Fig. 1A and B). The pronotum (Fig. 1D) was expanded with fine hairs that were shorter than the width of the eye. The labrum (Fig. 1D) was at the extreme anterior margin of the head, had 4-segmented antenna (Fig. 4C), had less than 11 recognizable segments on the abdomen (Fig. 1B) and a proboscis length that reached the middle of the first coxa (Fig. 1C).

### Socio-demographic profile of the respondents

The results showed that 50.2% of the 900 respondents were females compared to 49.8% males. The minimum and maximum ages were 18 and 96 years, respectively, with a mean age of 38.15 years. Overall, 84.2% of the households consisted of less than three occupants while 15.7% had three occupants and above (Appendix B, Fig. S1). This was based on a principle that one must have lived in the household for at least three months prior to the interview. About 58.9% of the respondents indicated that they had access to basic amenities in < 30 min, while the rest, 41.1% did not (Appendix B, Fig. S1).

### Bedbug perceptions and incidence in the communities

Most of the respondents could identify bedbugs from other known ectoparasitic arachnids like ticks, fleas, mites, and jiggers. About 80% of the respondents indicated bedbug infestation as a weird scenario, unlike the rest (20%) who perceived it as a normal occurrence in the society. In addition, more than 97% of the respondents affirmed that the pest is most active at night. When asked to indicate the severity of the bites, most respondents (68.9%) reported them to be mild, 28.8% said they were severe while 2.3% considered the bites side effects free. Those who experienced severe bites had to seek medical attention either by over-the-counter medication or at a medical facility. It was reported that among the recommended and received medication, anti-allergies, painkillers, and anti-inflammatory drugs were the commonest (Appendix B, Table S4). Out of the 900 respondents, 89.8% reported having disinfested their households more than once compared to 10.2% who did it once a year. Besides, bedroom/mattresses (34%), furniture (28%), cracks/crevices (24%), and clothes (14%) were the most infested household areas.

### Bedbug management practices

**Figure 2:**
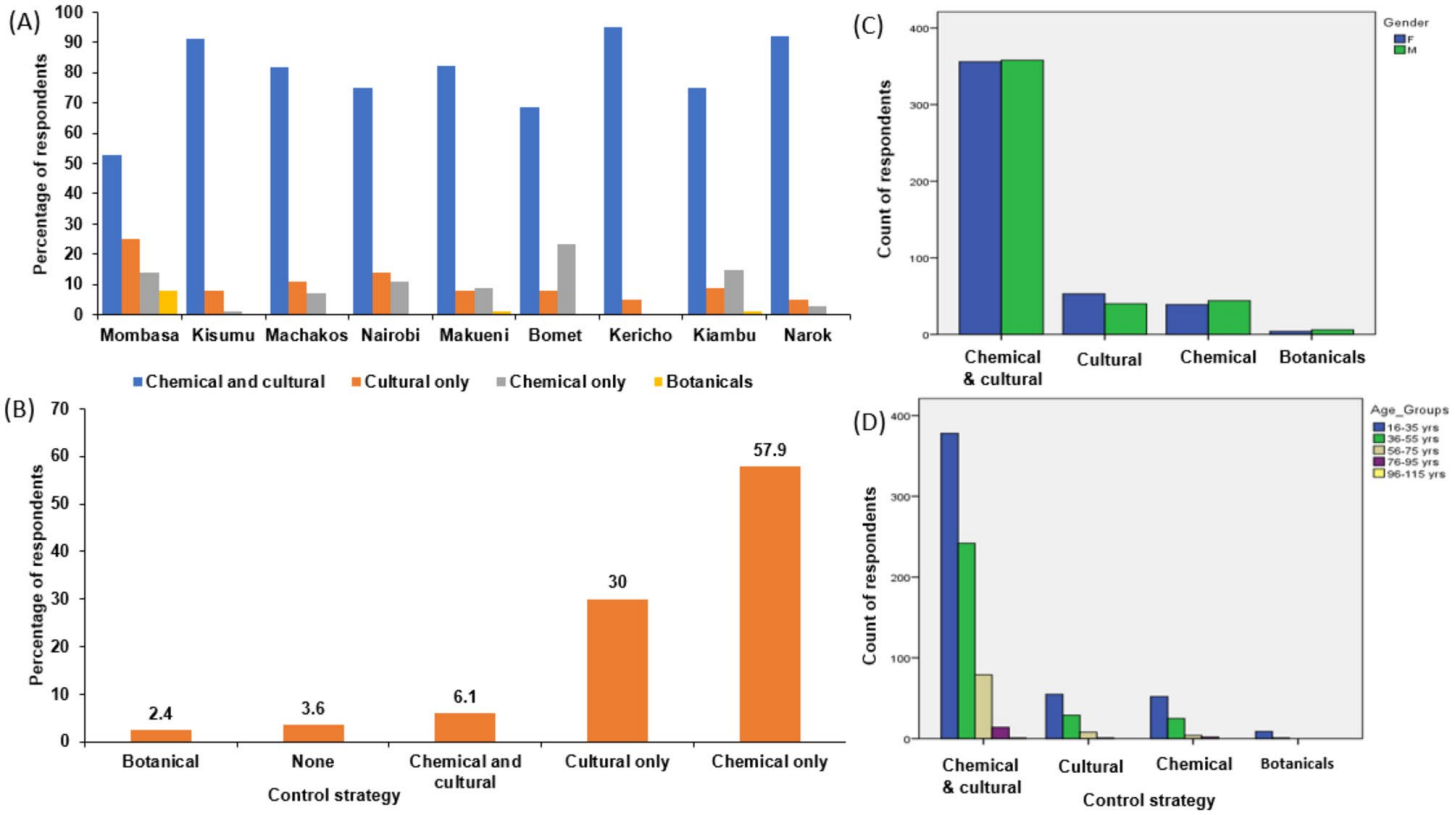
Control strategies recorded across different counties (**A**), the effectiveness of control and management practices (**B**), perception based on gender (**C**) and age group (**D**).

The residents reported having applied various bedbug control methods. These were generally grouped as; chemical and cultural, chemical only, cultural only, and botanical methods. Most respondents (79.3%), confirmed to have used both chemical and cultural practices, followed by cultural only 10.3%, chemical only 9.2% and botanical 1.1% (Fig. 2A). Besides, the applied cultural practices included use of hot water, exposure to sunlight, paraffin, diesel, and detergents while botanicals comprised the use of aloe vera extract and herbs (waragi). There was a statistically significant difference between the various control methods across the sampled counties (*X*^2^ = 145.226; *df* = 24, P ≤ 0.000) (Fig. 2A). However, when asked about their effectiveness, the use of chemical methods (pesticides) dominated with 57.9% followed by cultural methods at 30%, combined use of chemical and cultural methods at 6.1%, and use of botanicals was the least with 2.4%. Furthermore, only 3.6% of the respondents reported that none of the control strategies was effective enough (Fig. 2B). A significant statistical difference was detected in the level of effectiveness between different control practices across the surveyed counties (*X*^2^ = 495.555; *df* = 32, P ≤ 0.000). A total of 21 pesticides belonging to different classes of WHO classification were reported, where the most commonly/frequently used across the counties contain chlorpyrifos, carbaryl, cipronil, pyrethrin, imidacloprid, and gichlorvos as the active compounds (Appendix B, Table S4). Across gender and age categories, choice and preference of the control strategies was statistically insignificant (*X*^2^ = 2.506; *df* = 3, P ≤ 0.04 and *X*^2^ = 11.109; *df* = 12, P ≤ 0.05 respectively).

Male respondents were reported to have been more likely to use combined chemical and cultural methods, chemical only, and botanical control methods than their female counterparts who preferred cultural management practices. Chi-square analysis exhibited that there was no significant difference between respondents in terms of gender (*X*^2^ = 2.506; *df* = 3, P ≤ 0.474) and age category (*X*^2^ = 11.109; *df* = 12, P ≤ 0.520) as regard to the choice of control strategy (Fig. 2C and D).

### Infestation dynamics of bedbugs in residential communities

**Figure 3:**
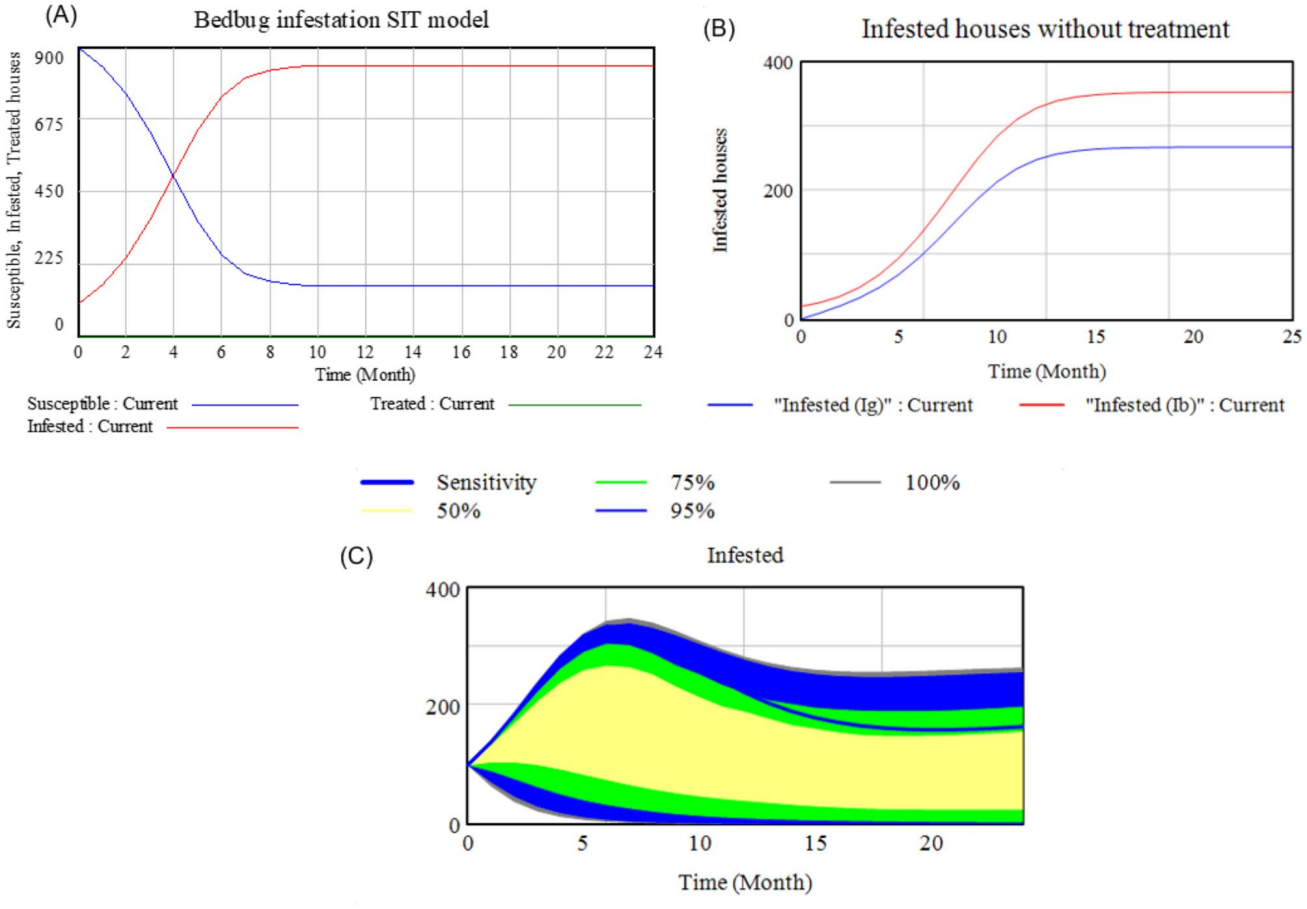
Infestation dynamic in homogeneous houses (**A**), in heterogeneous houses (**B**) communities and sensitivity test of the model (**C**).

The outcomes of the model showed that the infestation marginally increased while the number of susceptible houses decreased at the same rate and the equilibrium was reached after four months (Fig. 3A). Both dynamic curves reached a plateau after 10 months of simulation where about 94% of houses in the community were infested in the scenarios of homogeneous houses. When houses were categorized into bad houses (ancient houses with slits/fissure/crevices filled with old or secondhand furniture) and good houses (new houses or houses with less damage filled with new or reasonable quality furniture), the infestation in the community increased with a smooth slope and reached the plateau with some delay showing 15 months of simulation. Furthermore, the level of infestation for good houses (Ig) was much lower as compared to that of infested bad houses (Ib), translating into 42% and 58% of infestation, respectively (Fig. 3B). The sensitivity test showed that bedbug infestation among the community is highly influenced by the level of mobility of individuals in the population (Fig. 3C). For instance, the infection rate tends to increase when mobility level is high (Fig. 3C).

### Control strategies performance

**Figure 4:**
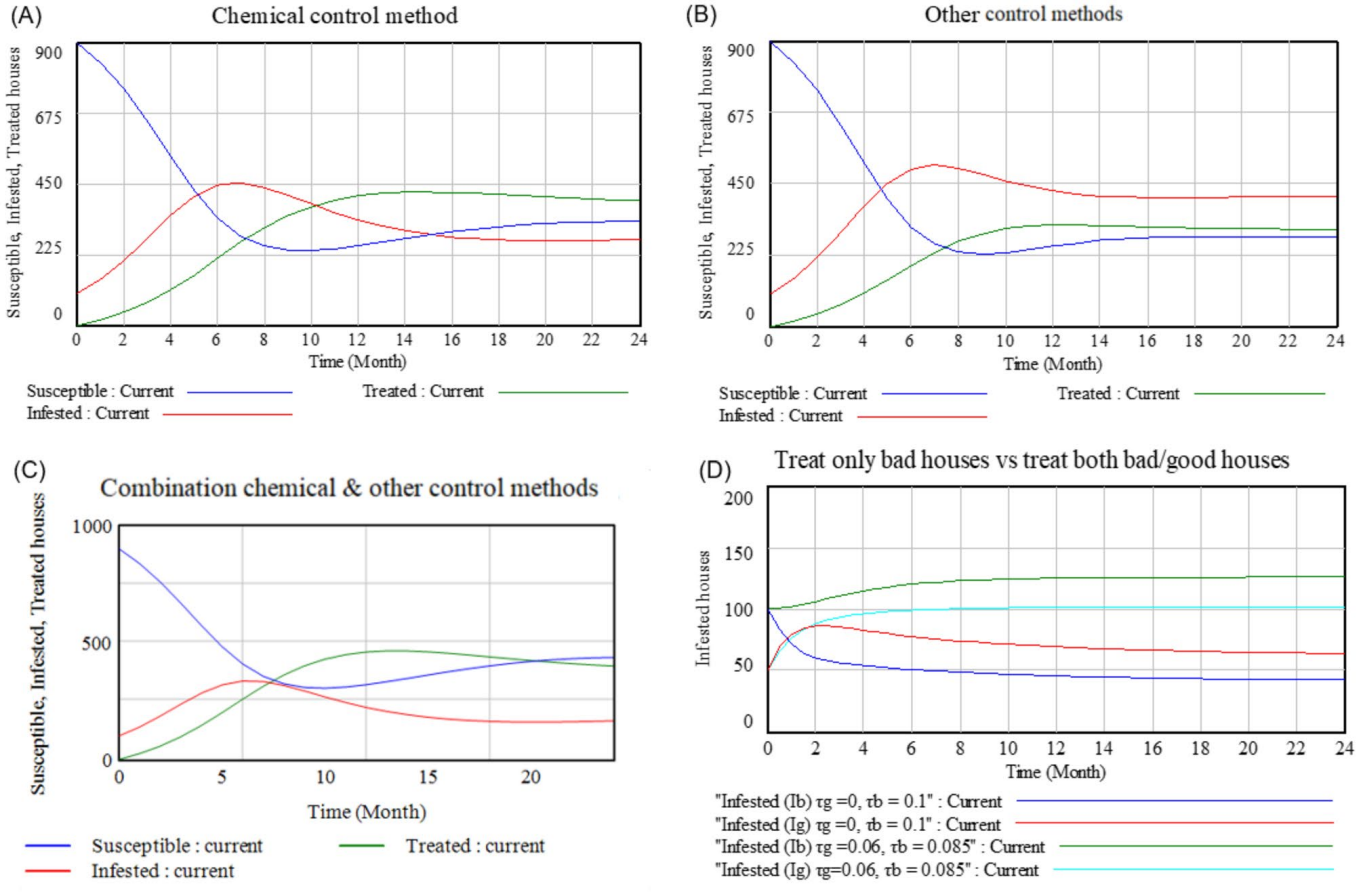
Control methods performance and management strategies.

A simulation considering the use of chemical control method (insecticides) showed that the number of susceptible houses decreased to approximately 300 in the first eight months (Fig. 4A). Also, the peak of infested houses was reached after seven months, translating to around 450 houses. Further, the peak of treated houses was reached after 14 months, translating to approximately 400 houses (Fig. 4A). Additionally, our SIT model was further simulated to understand the effects on using other control methods. The number of susceptible houses decreased drastically to around 225 houses in nine months while that of infested houses increased to approximately 500 houses in a period of eight months (Fig. 4B). The peak of treated houses was reached after 12 months translating to around 300 houses as well (Fig. 4B). When combining chemical method and other control methods, it was noted that an equilibrium involving susceptible, infested, and treated houses was reached after eight months translating to around 300 houses (Fig. 4C). Additionally, the peak of infested houses was reached after six months translating to approximately 300 houses, while that of the treated house was reached after 12 months translating to around 450 houses (Fig. 4C). It was noted that the use of other control methods for disinfestation was less effective as compared to the other scenarios (Fig. 4B). In the scenarios of heterogeneous houses within the community, focusing on the treatment of only bad houses with treatment rate of τ_b_ = 0.1 give a better result in reducing the number of infested houses than treating both good and bad houses with τ_g_ = 0.06 τ_b_ = 0.085, respectively (Fig. 4D).

### Potential distribution of bedbugs in Kenya and in Africa

**Figure 5:**
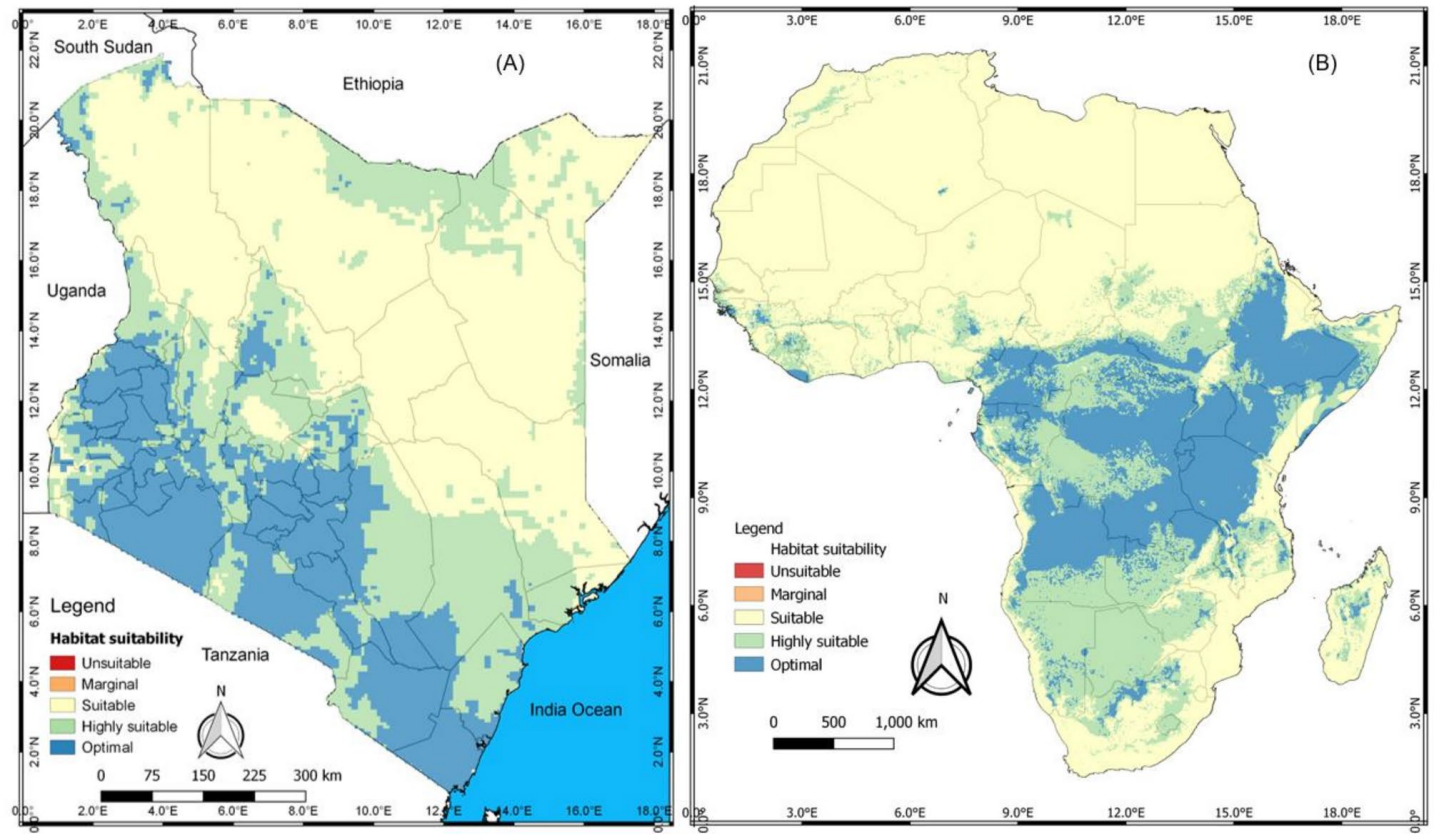
Potential geographical distribution of bedbug in Kenya and in Africa using the QGIS 3.10.2 software (https://qgis.org/downloads/).

The output map of Kenya showed regions with optimal to suitable for bedbug occurrence in the whole country (Fig. 5A). No unsuitable area of the pest occurrence was predicted across the country. The eastern and central regions of the country were more optimal while other regions varied from marginal to suitable zones and highly suitable zones could be found here and there across the country (Fig. 5A). Similar results were found at continental level varying from marginal to optimal occurrence zones across Africa (Fig. 5B). Eastern and central African countries were more optimal with highly suitable zones in southern countries while it can be found here and there in West-African countries.

### Bedbug *Cimex* spp. habitat suitability model

**Figure 6:**
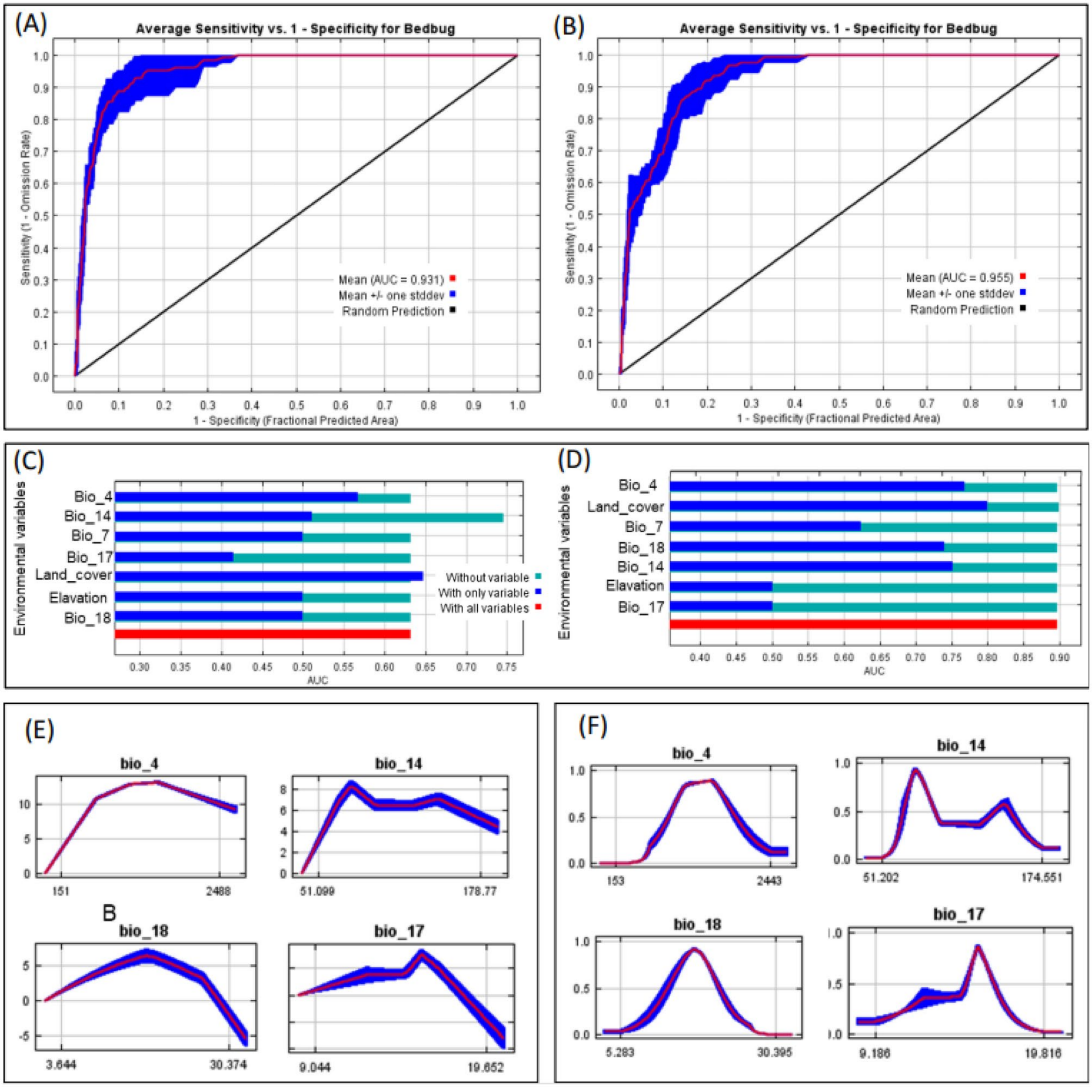
Receiver Operating Characteristic (ROC) curve verification of the predicted potential habitat for bedbug in Kenya (**A**) and in Africa (**B**); the relative importance of environmental variables for predicting the suitability habitat of bedbugs in Kenya (**C**) and Africa (**D**) and response curves between the probability and environmental variables in Kenya (**E**) and in Africa (**F**).

The AUC values were all higher than 0.6, indicating the optimal occurrence area of the pest (Fig. 6A and B). This shows that the model successfully predicts the suitable habitat of the bedbug in Kenya and in Africa.

The jack-knife test demonstrated that land cover and Temperature Seasonality (bio_4) are the most important variable in determining the suitability of bedbug in Kenya (Fig. 6C); while in addition to the lates, Precipitation of Driest Month (Bio_14) and Precipitation of Warmest Quarter (Bio_18) variables significantly contribute to determining its suitability in Africa (Fig. 6D). In both cases, four bioclimatic variables: Bio_4, Bio_14, Bio_18 and Bio_7 (Temperature Annual Range (BIO5-BIO6)) have at least 0.5 value AUC, which indicated that they were the main bioclimatic factors affecting the potential distribution area of the bedbugs. The response of bedbugs to those four bioclimatic variables in Kenya is presented in Fig. 6E. In Africa, the distribution probability of the pest increased with the increase of the value of each bioclimatic variable within a certain range and decreased with the increase of the variables after a certain peak value (Fig. 6F).

## Discussion

Community-wide perception and knowledge assessment on bedbugs revealed an ample awareness of the population and several interesting bedbug infestation patterns. The respondents have confirmed to experience a resurgence of bedbug infestations and had high infestation rate. Bedbug resurgence and infestations have recently been reported globally as a significant health problem^25^. In 2018, Kenya reported 4000 bedbug-infested homes, therefore the rise of infestations parallels that with other African nations and the entire world^27^. Moreover, the pest has been reported as a potential public health hazard and a socioeconomic burden with approximately USD 2500–3000 USD per infestation spent in disinfection and replacement of infested belongings^24^. The 50.2% of females versus 49.8% of male respondents recorded during the survey of this study was an important indicator to avoid biasness of gender in the variables of our data.

The high prevalence of bedbugs is directly related to psycho-socio-economic-health and hygienic effects. Overall, 80% of the respondents during our survey perceived bedbugs as a weird occurrence as compared to 20% who perceived them as a normal pest in society. This is because the pest is associated with poor economic status and an unhygienic environment^28^. However, bedbugs affect all socioeconomic backgrounds with no poverty level correlation^29^. This phenomenon can be attributed to various factors including infestation in public transport such as airplanes, ships^3^ and vans^30^. Besides, human habitats, especially the permanent ones like residential premises, serve as potential reservoirs for the ectoparasites. In contrast, public accommodation acts as converging and diverging points for new infestations. Nearly 69% of mild bite symptoms were reported as predominant across the study areas. This could have been due to a lack of inflicted pain and immediate signs since a delay of the response of up to nine days has been documented in the past^31^. On the other hand, 29% severe bite reactions due to high sensitivity are reported to have been treated by visiting a medical facility or acquiring antiallergics or painkillers over the counter. The results of our study concur with other studies, that have documented the use of oral antihistamines, topical corticosteroids, and topical emollients to treat bedbug bites^2^. Furthermore, not always all the bites result from bedbugs, so every bite should be confirmed. Bedroom/mattresses, furniture, cracks/crevices, and clothes were the most infested areas in the infested houses. This kind of distribution is probably due to frequent dispersing since the ectoparasite establishes new harborages once the habitat does not favor them anymore^32^. Gangloff-Kaufmann et al.^33^ and Hwang et al.^2^ have reported bedbugs to mainly inhabit mattresses and beddings. In a resource-limited setting, it is quite obvious that the economically and socially disadvantaged groups share a significant burden of infestation due to limited or no financial capabilities to disinfest. This was observed in our study where some respondents disinfected their habitat/materials once per year and some could not even afford the cost of the treatment.

In order to understand the dynamics of bedbugs infestation in residential community, this study used a simple metapopulation approach and proposed SIT model by having two cases: a basic model for homogeneous houses and advanced model for heterogeneous houses. The infestation dynamic and interactions of bedbug species in a residential community model showed a rapid mobility of bedbug from one house to another where about 94% of houses in the community were infested after 10 months post introduction of the pest in the community. Bedbugs do not fly since they do not have wings. They are only able to crawl and move short distances within an infected area, and slowly spread to other rooms in the house. Therefore, the high or rapid spread of the pest in the community is mostly attributed to human factors. Bedbugs then rely upon humans to help transporting them to new areas and locations where they are free to set up and establish new infestation. People get bedbugs from being at places where bedbugs are, as they are hitch-hikers and largely dependent upon human mobility to travel from one place to another. Bedbugs can climb into luggage or other belongings and are then brought home. They can also move around by finding their way into purse, backpacks, clothing, suitcases, briefcases, and jackets and one can carry them to other locations by simple visit of infested neighbouring house. When considered the scenarios of heterogeneous of bad and good houses, the number of infested good house (Ig) was much less as compared to the infested bad houses (Ib). This could be explained by the fact that low income, less education, unemployment, living in group homes and staying at homeless shelters are perceived as some of the factors attributed to bedbug infestations^34^. This confirmed the hypothesis that new or improved houses labelled as “good” houses do not provide very suitable conditions for the survival of bedbugs.

Further, our spatial distribution analysis of bedbugs in Kenya and in Africa results showed that the habitat suitability of *Cimex* spp. (Hemiptera: Cimicidae) predicted for the current scenario agreed strongly with the ground observations. According to Koppen-Geiger climate classification^35^, the tropical climate has been grouped into three including: tropical rainforest, tropical monsoon and tropical savannah. In Kenya, this climate is mainly found along the coastal region and lakeside (Lake Victoria), although fragmented in some other parts of the country. Our distribution prediction results are in tandem with this classification since *Cimex* spp. habitat suitability has been predicted as optimal and highly suitable in these regions. Temperate climate, which covers a relatively larger part of Kenya (central, western, and southern), has been predicted as areas of optimal and high habitat suitability for the pest. Also, the northern and northeastern parts of Kenya, usually known to be semi-arid, have been predicted as a suitable habitat for *Cimex* spp. in our study. In general, our study demonstrated Kenya as entirely a potential suitable habitat for *Cimex* spp. since none of its regions has been predicted as marginal or unsuitable habitat. Furthermore, our prediction across Africa concurred with Koppen-Geiger climate classification^35^. The northern arid and semi-arid part of Africa has been predicted as a suitable habitat for the pest. Tropical climates, found in central and western Africa regions, have been predicted to have optimal habitat suitability. However, some regions in this area have been predicted as highly suitable for bedbugs. Finally, southern Africa, which has a subtropical and temperate climate, has been predicted to be a region of suitable and highly suitable habitats. Therefore, this study delineates Africa as a potential habitat of *Cimex* spp. (Hemiptera: Cimicidae) as well.

The habitat predicted by our model is similar to that of the known occurrence point; however, there was some observed deviations from the actual occurrence areas of the pest. Besides the bioclimatic variables as initial predictors, other non-climatic factors such as human activities, control measures, host and natural enemy affect species habitat suitability^36^. Generally, models are just an estimate of a species potential distribution and cannot replace fieldwork but rather can be an indispensable tool for data exploration meant to help identifying knowledge gaps and provide guidance for fieldwork design^37^.

As the pest became a serious public health nuisance with its high population dynamics and high potential geographical distribution, bedbug merits serious management approaches. Residents have reported to have applied various control and management practices. This comprised use of chemical method, cultural method, and botanicals but respondents highlighted chemical method as the most effective. Furthermore, the model simulations' outputs indicated that use of chemical control method was more effective than using other control strategies. This can be attributed to the fact that insecticides are reported as a powerful weapon in eradicating domestic pests and fighting against other vector diseases, as well as the existence of a broad range of insecticides, including pyrethroids, neonicotinoids, pyrroles, organophosphates, and carbamates^38–40^. In our study, some of the predominant insecticides used across the surveyed counties contain Chlorpyrifos, Carbaryl, Fipronil, Pyrethrin, Imidacloprid, and Dichlorvos as active compounds. According to the World Health Organization (WHO), Chlorpyrifos 480 g/l, Carbaryl 7.5%, Fipronil, 6% Pyrethrin and Imidacloprid 200 g/l, all have been classified as class II (moderately hazardous) while Dichlorvos 1000 g/l as class 1b (highly hazardous)^41^. Therefore, the haphazard and unsafe use of those pesticides could result in serious negative effects on the environment, human and animal health. In addition, most of the residents are reported to use higher concentrations of these pesticides than stipulated and not adhering to the label rates. This puts them at an increased risk of indirect health problems such as toxicological health issues^25,42^. Furthermore, insecticide overuse has resulted in ever increasing organophosphate concentrations in water bodies^43^, which puts the residents at a risk of chronic diseases such as asthma, kidney disorders, hormonal disorders and other insecticide-related toxicological disorders^25^. Due to the toxic nature of insecticides and unregulated application practices, appropriate awareness creation is very important to be conducted.

Fortunately, the outputs of model simulations showed that the combination of chemical methods with other control methods was considerably much more effective in reducing the number of infested houses in the community compared to even the use of chemical method only and concentrating on the treatment of only bad houses was the better option. Therefore, integrated pest management (IPM) strategy by combining different control methods should be a better intervention approach in controlling bedbugs as it also limits dependence on pesticides and reduces risks for humans and the environment. Cooper et al.^44^ have reported that adopting a complex-wide bedbug IPM program, proactive monitoring, and biweekly treatments of infested houses utilizing non-chemical and chemical methods can successfully reduce infestation rates to very low levels.

## Conclusion

Bedbugs, a voracious blood-feeding ectoparasite, is indeed a potentially significant health problem within resident communities in Kenya. This calls for rapid action, immense awareness campaigns accompanied by technological innovation and national, regional, and international cooperation to develop a long-lasting, suitable and sustainable solution. This study successfully modelled the mobility and infestation dynamics patterns for *Cimex* spp. It also provides the first predicted potential habitat distribution map for the bedbug species in Kenya and Africa. MaxEnt might have over predicted in some areas; however, the information provided in our study is timely and highly relevant given the current frequent outbreaks of bedbugs, as well as the threat of *Cimex* spp. as a potential health hazard to the public. The predicted potential habitat distribution map of *Cimex* spp. could therefore help in planning, monitoring, and identifying top-priority survey sites; and integrated pest management approach is highly recommended in the management of the pest as the model showed that the combination of chemical methods with other control methods was considerably much more effective in reducing the number of infested houses and the pest pressure in the community compared to the use of chemical methods only.

## Materials and methods

### Sample collection

A survey was conducted among the residents of nine counties in Kenya (Mombasa, Kisumu, Machakos, Nairobi, Makueni, Bomet, Kericho, Kiambu, and Narok) and GPS location coordinates were recorded and later used to build the predictive model (“Infestation dynamics of bedbugs in residential communities” section). These counties represent diversity in cultural practices, livelihood strategies (such as fishing, tourism, farming), and infrastructure development. Also, they comprise different altitudes above sea level, temperatures, and differing in average annual rainfall.

### Samples identification using morphological identification keys

In each county where the survey was conducted, bedbug samples was taken and preserved in ethanol 70% for morphological identification. Cimex belonging to Cimicidae family is the common genus adapted to human environment and reported throughout the world and comprising species such as *Cimex lectularius* and *C. hemipterus* that are hematophagous mainly feeding on human blood^5^. The key morphological features used in identifying bedbugs include: (1) the head has a labrum that appears as a free sclerite at the extreme anterior margin, ecdysial lines form a broad V, eyes project from the sides composed of several facets and the antennae are 4-segmented, (2) thorax is subdivided into prothorax, mesothorax and metathorax, (3) legs have all other normal parts except pulvilli and arolia, tarsus is 3-segmented with 2 simple claws, (4) the abdomen has 11 more-or-less segmented recognizable segments, 7 pairs of spiracles borne on the second to eighth segments, hosts the genital structures, paramere in males and mesospermalege in females^45^. Bedbug specimen morphological features were examined using Leica EZ24 HD dissecting microscope (Leica Microsystems, UK) and photos documented using the associated software.

### Survey for household’s knowledge and perceptions on bedbugs

This study was a community-based cross-sectional survey conducted from November–December 2020 with respect of the rules/guidlines introduced by the Ministry of Health to contain the COVID-19 pandemic in Kenya (wearing mask, social distance, washing hand, etc.). It was based on a stratified, systematic random sampling where 100 respondents were selected from each county.

A total number of 900 respondents were randomly selected and the household head or the representative showing willingness and consent was interviewed face-to-face. The interview was conducted using a semi-structured questionnaire prepared in the English language (Appendix A). The questionnaire was translated into the local native language (Kiswahili) to avoid biasness and improve the understanding between the enumerator and the respondent. Prior to the commencement of the survey and authentic data collection, a pre-testing exercise was performed by training enumerators on a similar socio-demographic pattern. This was useful for improving the quality of data, ensuring validity, familiarizing the enumerators with the questionnaire, and data handling.

The information collected using the semi-structured questionnaire included residents’ socio-economic profiles, knowledge, and perceptions on the pest, bedbug incidence, and management practices. The socio-economic profile factors addressed in the survey comprised gender, age, education, access to basic social amenities, and household size. The study also prioritized the financial consequences, the severity of the bites, perceptions of respondents on the pest, and management practices for its control.

Survey data were checked for errors, completeness, summarized, and entered in Microsoft-Excel. It was then cleaned and transferred to Statistical Package for Social Science (SPSS) version 25 software (IBM Corp., Armonk, NY) for purposes of descriptive statistics (means and percentages).

In contrast, in instances where more than one reason was given for a single question, percentages were calculated based on each group of similar responses. Chi-square was performed to determine the differences regarding socio-demographic characteristics, knowledge, and perceptions on bedbugs and control practices. Additionally, data were disaggregated by gender and age categories to understand the existing differences among the various respondent categories. Besides, F-test statistics was performed on the ages of respondents to determine the mean, standard deviation and statistical significance. The level of significance was considered when the p-value was below 5%.

### Infestation dynamics model of bedbug

#### Model simulation assumptions

Houses infestation dynamics was studied following Susceptible-Infested-Treatment (SIT) model^46^. Therefore, houses in the community are classified into three groups: susceptible, infested or treated. Within a house, bedbug population dynamics was ignored, while it was considered from one house to another where infested houses have some potential to spread the infestation to other houses in the community. A population of bedbugs in an infested house has some probability per unit of time of becoming extinct either naturally or after treatment. In the infestation dynamics, the rate of house infestation depends on the number of infested houses, the movement of people from one house to another and the proportion of treated houses in the community. We assume that infested houses (I) spread the infestation at the rate β and only a fraction S/N of the houses is susceptible (S) to infestation. Infested houses become extinct at a certain rate known as rate γ. Infested houses are treated at the rate τ and the protection conferred is lost at the rate α. Ordinary differential equation developed to study SIT model were used in this study^46^. All the models used have the generic formulations displayed below:

1 $$\frac{dS}{dt}=\frac{\beta }{N}SI+\gamma I+\alpha T$$

2 $$\frac{dI}{dt}=\frac{\beta }{N}SI-(\gamma +\tau )I$$

3 $$\frac{dT}{dt}=\tau I-\alpha T$$

where β > 0, τ > 0, α ≥ 0 and γ > 0. The total population size is N = S(t) + I(t) + t(t). The initial conditions satisfy at S(0) > 0, I(0) > 0, T(0) ≥ 0 and S(0) + I(0) = N, where N is the constant total population size, dN/dt = 0.

#### Infestation dynamics models implementation

The method used to implement the infestation dynamics model of the pest is based on the system thinking approach with its archetypes [Causal Loop Diagram (CLD), Reinforcing (R) and Balancing (B)] by a mental and holistic conceptual framework. This is important for mapping how the variables, issues, and processes influence each other in the complex interactions of bedbugs within and between houses and their impacts. Despite these archetypes being qualitative, they are necessary for elucidating and disclosing the basic feedback configurations that occur in houses and their environs when infested with pests like bedbugs. A dynamic model was generated by converting the causal loop diagram (CLD) obtained using stocks, flows, auxiliary links, and clouds. Consequently, these in turn were translated into coupled differential equations for simulations.

The SIT model was translated into causal loop diagram where arrows show the cause-effect relations where positive sign indicates direct proportionality of cause and effect while negative sign shows inverse proportionality relations, and two different scenarios have been assessed: (1) homogeneous houses where there is a single community of houses of the same quality, and (2) heterogeneous houses where there is a community of good and bad houses. Ancient houses presenting slits/fissures with less cleanliness and filled with old or secondhand furniture at low grade are considered bad houses as they may sustain high level of bedbug infestation; and new houses don’t provide well enough conditions for bedbug population to survive, and they are called in the model good houses^47^. Bad houses are considered to act as sources while good houses act as sinks, but all together are randomly distributed where each house has the same probability to contact good or bad houses.

**Figure 7:**
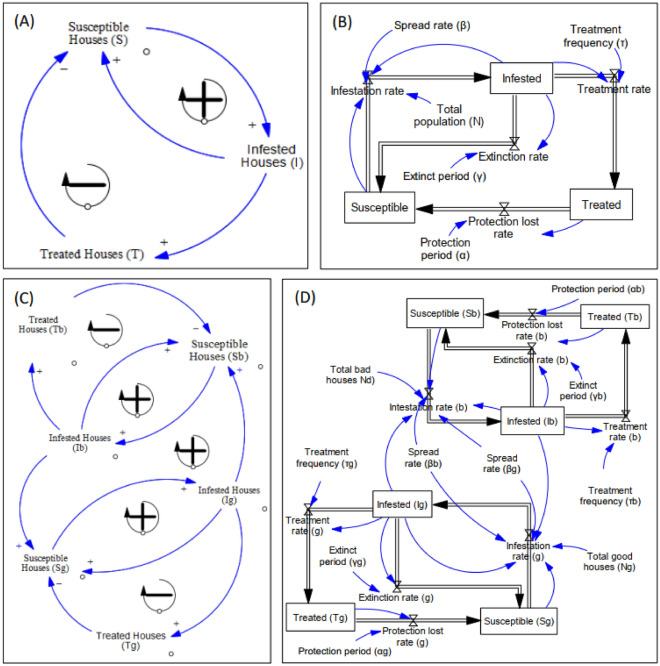
Susceptible-Infested-Treatment (SIT) model translated into causal loop diagram (**A**) and stock and flow diagram (**B**) for homogeneous houses and causal loop diagram (**C**) and stock and flow diagram (**D**) for heterogeneous houses in the community.

In the scenarios of homogeneous houses, the causal loop diagram (Fig. 7) has two feedback loops: (a) one positive, as the number of infested houses increases, the probability to get susceptible houses infested also increases resulting in infested houses increase; (b) one negative, as the infested houses increases, the treated houses increase resulting in susceptible houses decrease. The causal loop diagram is displayed in Fig. 7A while Fig. 7B showed the stocked and flows diagram and axillary variables obtained from causal loop diagram.

Susceptible, infested, and treated houses are stocks in the system, representing the number of houses susceptible, infested, and treated, respectively at a given point of time. The rates represent in and out-flows of the diagram. Auxiliary and constants that drive the behavior of the system were connected using information arrows within them and flows and stocks to represent the relations among variables in terms of equations.

In the scenarios of heterogeneous houses, the causal loop diagram (Fig. 7C) comes with the two previous feedback loops but for each category of house. In addition, there is a fifth feedback loop that connect bad house to good house and vice versa.

Therefore, as the infested bad houses increase, the probability to infest good houses increases. The more they are exposed the more they get infested. In turn, as the infested good houses increase, the chance to infest susceptible bad houses increases and the more they are exposed, the more they get infested, resulting in the increase of infested bad houses. The stocks and flows diagram of each of the two categories of houses occurred with interconnexion relationships between the two categories (Fig. 7D).

#### Models’ simulations

The survey data (“Bedbug Genus identification” section) on prevalence, knowledge, perceptions and self-reported; in addition, the respondents' reported control mechanisms and their average time of effectiveness (Appendix B, Table S1) were used for model simulations. The different control methods reported were reclassified in three control approaches: chemical control, other control methods (including exposure to direct sunlight, use of hot water, painting, application of diesel, paraffin and wood ash, use of Aloe Vera extract and Herbs), and combination of chemical and other control methods. All the models commodities and units were checked before performing the simulations. Simulation and implementation of the models were done using Vensim PLP 8.1 platform (Ventana systems, Harvard, USA). It consists of a graphical environment that usually permits drawing of Causal Loop Diagram (CLD), stocks, flow diagrams and to carry out simulations. After we simulated the infestation dynamics under the two scenarios, we explored the effect of the different control methods.

### Spatial distribution analysis of bedbugs using MaxEnt model

#### Environmental data for MaxEnt

The environmental variables used as the other maxent input were obtained by deriving bioclimatic, land cover, and elevation data. Bioclimatic variables and elevation (Digital Elevation Model; DEM) data were obtained from the Global Climate Data official website, Worldclim (http://www.worldclim.org/bioclim.htm)^48^ including 19 bioclimatic variables (Appendix B, Table S2). The land cover data were downloaded from the Global Land Cover Facility (GLCF).

**Figure 8:**
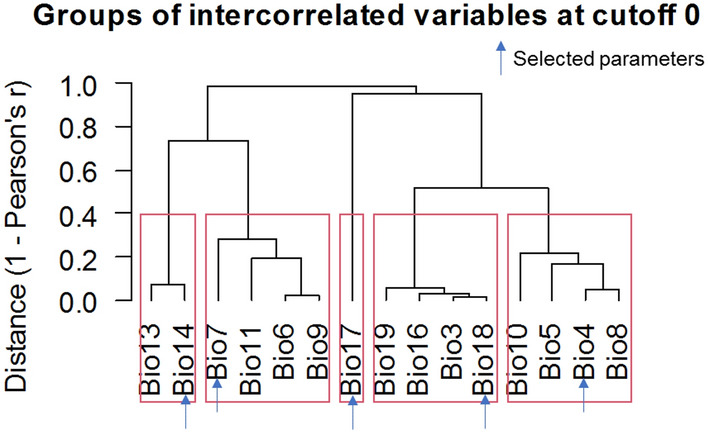
Key model predictor variables.

In order to reduce collinearity between predictors, a collinearity test was performed on all the variables by filtering them according to the following steps^36^: firstly, the MaxEnt model was run using the distribution data of bedbugs and 19 bioclimatic variables to obtain the percent contribution of each variable to the preliminary prediction results. Secondly, following the generation of the percentage contribution of all the variables, we then imported all distribution points in Arc-GIS and extracted the attribute values of the 19 variables. Furthermore, the “virtual species” package^49^ in R-software (R Foundation for Statistical Computing, Vienna, Australia) was used to explore the extracted variables' clusters spatial correlation using Pearson's correlation coefficient and the cluster tree (Fig. 8). Thus, the final number of predictor variables after screening was 5 establishing the potential geographical distribution of bedbug, which includes Temperature Seasonality (bio4), Precipitation of Driest Month (bio14), Temperature Annual Range (bio7), Precipitation of Driest Quarter (bio17) and Precipitation of Warmest Quarter (bio18) (Appendix B, Table S2). The land cover was considered because studies have shown its importance on insect spatial distribution^50–52^ and it was setled as a categorical variable^53^. Elevation was selected as variable because it greatly influences species' occurrence and dispersal by affecting the temperature, precipitation, vegetation, and sun characteristics (direction, intensity, etc.) on the earth's surface^54–56^. The study variables had different resolutions and were therefore, resampled to 1 km. The variables were clipped to Kenya and Africa boundaries and converted to ASCII (Stands for "American Standard Code for Information Interchange”) format using the 'raster' package^49^ in R statistical software (R Foundation for Statistical Computing, Vienna, Australia).

#### Distribution modelling in Kenya and Africa

In our study, we used the maximum entropy distribution modelling method. This is because it has been recommended to have the ability to perform best and remain effective despite the use of small sample size relative to the other modelling methods^57^.

Our selected bioclimatic variables (5) and occurrence/prevalence data for bedbugs were then imported into MaxEnt model and the options of ‘Create response curves’ and ‘Do jackknife’ were selected to measure variable importance’ options. The model output file was selected as ‘Logistic’, the commonly used approach is the random portioning of distribution datasets into ‘training’, and ‘test’ sets^57,58^. MaxEnt model was run with a total number of 5000 iterations and five replicates for better convergence of the model and rescaled within the range of 0–1000 suitability scores using 'raster' package^49^ in R statistical software (R Foundation for Statistical Computing, Vienna, Australia).

The modelling performance/MaxEnt accuracy was evaluated by choosing the area under the receiver operating characteristics (ROC) curve (AUC) as the estimation index. This was important for the calibration and validation of the robustness of MaxEnt model evaluation. Furthermore, the area under the ROC curve (AUC) was necessary as an additional precision analysis^59^. The range of AUC values greater than 0.7 was considered a fair model performance, while those greater than 0.9 indicated that the model was considered an excellent model performance. Therefore, by considering the AUC values, the excellently performing model was selected to analyze the suitability of bedbugs in Kenya and Africa^59–62^.

The ASCII format output was then imported into QGIS 3.10.2 (using the QGIS 3.10.2 software, https://qgis.org/downloads/), following its conversion into a raster format file using R software. This was useful for the classification and visualization of the distribution area^63,64^. The potential suitable distribution of bedbugs was extracted using the Kenyan and African maps. At the same time, Jenks' natural breaks were also used to reclassify and classify the suitability into five categories, namely: unsuitable (P < 0.2), marginal (0.2 < P < 0.4), suitable (0.4 < P < 0.6), optimal (0.6 < P < 0.8) and highly suitable (P > 0.8) area^36^. Therefore, MaxEnt modelling was used to predict the distribution of *Cimex* spp. (Hemiptera: Cimicidae) in Kenya and Africa using our collected geo-referenced occurrence records.

### Ethics approval

The experimental research and field studies on plants, including the collection of plant material, complied with relevant institutional, national, and international guidelines and legislation. The appropriate permissions and/or licenses for collection of insect, plant or seed specimens were obtained for the study. All insect handlings were performed using standard operating procedures at the *icipe* Animal Rearing and Quarantine Unit as approved by the National Commission of Science, Technology and Innovations, Kenya (License No: NACOSTI/P/20/4253). This article does not contain any studies with human participants performed by any of the authors.

## Supplementary Information

Supplementary Information.

## Data Availability

The dataset generated during the current study are available from the corresponding author upon request.
