# Supplementary material for: Household perception and infestation dynamics of bedbugs among residential communities and its potential distribution in Africa
Source: Sci Rep. 2022 Nov 18;12:19900. doi: 10.1038/s41598-022-24339-7 (PMC9674637; doi:10.1038/s41598-022-24339-7)
Supplement: Supplementary file 1 — Supplementary Information. [file 41598_2022_24339_MOESM1_ESM.doc]

**Appendix A**

**QUESTIONNAIRE MODULES FOR THE ASSESSMENT OF BEDBUGS OUTBREAKS, DISTRIBUTION, MANAGEMENT AND CONTROL PRACTICES AMONG RESIDENTS IN DIFFERENT COUNTIES IN KENYA.**

| **Introductory statement:**  “Dear Sir/madam, I work for the International Centre of Insect Physiology and Ecology (*icipe*). We are conducting a survey to study the level of infestations, economic impact, perceptions, knowledge, public awareness and practices regarding bedbugs outbreaks, distribution, control and management around the country. Your response to these questions would remain anonymous, and taking part in this study is voluntary. We request that you answer the questions as accurately and honestly as possible so that our understanding and future activities/actions are then based on addressing the real pest control, management and problems faced by residents like yourself. If you choose not to take part, you have the right not to participate and there will be no sequences. We also promise to respect all the guidelines/barrier measures (wearing mask, social distance, washing hand, etc.) set by the Ministry of Health to contain COVID-19 disease in Kenya. Thank you for your kind co-operation.” |
| --- |

MODULE 1. HOUSEHOLD AND VILLAGE IDENTIFICATION

| **1.1 Household Identification** | **Code** |  | **1.2 Interview details** | **Code** |  |
| --- | --- | --- | --- | --- | --- |
| **1**. County | \|  \|  \| \| --- \| --- \| |  | **14**. Date of interview (dd/mm/yyyy): | \|  \|  \| / \|  \|  \| / \| 2020 \| \| --- \| --- \| --- \| --- \| --- \| --- \| --- \| |  |
| **2**. Sub-county | \|  \|  \|  \| \| --- \| --- \| --- \| |  | **15.** Time started (24 HR) |  |  |
| **3**. Ward | \|  \|  \|  \| \| --- \| --- \| --- \| |  | **16**. Name of enumerator |  |  |
| **4.** Location | |  |  |  | | --- | --- | --- | |  | **17**. Name of supervisor: |  |  |
| **5.** Village: |  |  | **18.** Name of data entry clerk |  |  |
| **6.** Age of respondent |  |  |  |  |  |
| **7**. Name of household head (three names): |  |  |  |  |  |
| **8**. Sex of household head  1=Male  0=Female | |  | | --- | |  |  |  |  |
| **9.** Name of the respondent (three names): |  |  | **GPS reading of homestead** | |  |
| **10.** Sex of respondent  1=Male  0=Female | \|  \| \| --- \| |  | **19.** Way point number |  |  |
| **11.** Name of respondent’s spouse |  |  | **20.** Latitude (North) |  |  |
| **12**. Cell phone number of household head   \|  \|  \|  \|  \|  \|  \|  \|  \|  \|  \| \| --- \| --- \| --- \| --- \| --- \| --- \| --- \| --- \| --- \| --- \| | |  | **21.** Longitude (East) |  |  |
| **13.**Cell phone number of the spouse:   \|  \|  \|  \|  \|  \|  \|  \|  \|  \|  \| \| --- \| --- \| --- \| --- \| --- \| --- \| --- \| --- \| --- \| --- \| | |  | **22.** Altitude (meter above sea level) |  |  |

MODULE 2: HOUSEHOLD COMPOSITION, CHARACTERISTICS AND HOUSING CONDITIONS

**2.1 HOUSEHOLD COMPOSITION AND CHARACTERISTICS** (*Household members: persons who live together and eat together from the same pot (share food), including hired labour, students and spouse living and working in another location but excluding visitors*)

| ID CODE | Name of household member  [Start with respondent] | Sex  1=M  0=F | Relationship to the household head  **CODE 1** | Age (complete years; 0 if less than 1 year) | Marital status?  **CODE 2** | Education (years)  **CODE 3** | Primary occupation  **CODE 4** | How many months in the past year was [NAME] present in the household? | Number of children living in the same household (under age of 18yrs) | Number of children living in the same household (above age of 18yrs) |
| --- | --- | --- | --- | --- | --- | --- | --- | --- | --- | --- |
|  | **AA1** | **AA2** | **AA3** | **AA4** | **AA5** | **AA6** | **AA7** | **AA8** |  |  |
| 1 |  |  |  |  |  |  |  |  |  |  |
| 2 |  |  |  |  |  |  |  |  |  |  |
| 3 |  |  |  |  |  |  |  |  |  |  |
| 4 |  |  |  |  |  |  |  |  |  |  |

| **CODE 1** | | **CODE 2** | **CODE 3** | **CODE 4** | |
| --- | --- | --- | --- | --- | --- |
| 1.Household head  2.Spouse  3.Son/daughter  4.Parent  5.Son/daughter-in-law | 6. Grandson/granddaughter  7.Other relative  8.Hired worker  9.Other, specify…………… | 1.Married living with spouse  2.Married living without spouse  3.Divorced/separated  4.Widow/widower  5.Never married | 0. None/Illiterate  1. Adult education or 1 year of education  * Give other education in years (e.g. 2 yrs for std 2, 8 yrs for class 8 etc)  100. Religious education | 1.Farming (crop+ livestock)  2.Salaried employment  3.Self-employed off-farm  4.Casual labourer on-farm | 5.Casual labourer off-farm  6.School/college child  7.Non-school child  8.Other, specify………….. |

**MODEL 3: BED BUG CONTROL AND MANAGEMENT PRACTICES**

3.1. Have you experience bed bugs menace/nuisance before?

1. Yes ( )
2. No ( )

3.2. If yes, for how long have you encountered bed bugs?

1. 1-5 years ( )
2. 6-10 years ( )
3. 10-15 years ( )
4. 16-20 years ( )
5. Above 20years ( )

3.3. What are some of the methods you have tried to manage them?

1. Burn with hot water ( )
2. Exposing infested items to sunlight outside ( )
3. Use of pesticides ( )
4. Any other specify ( )

3.4. Which of the above-mentioned methods has been the most effective?

3.5. On the above-mentioned effective method(s), was it a one-time treatment or on repeated occasions?

1. Once ( )
2. Repeated ( )

3.6. How many pesticides have you tried so far to eradicate the bugs?

3.7. On the above-mentioned types, which one would you recommend as the most effective pesticide?

1. None ( )
2. Any other specify ( )

3.8. After fumigation, how long does it take for the bugs to disappear?

1. 1 month ( )
2. 2 months ( )
3. 3 Months ( )
4. Above 4 months ( )

3.9. Have you ever relocated before because of the nuisance pest?

1. Yes ( )
2. No ( )

3.10. If YES, did it work for you?

1. Yes ( )
2. No ( )

3.11. How adverse are the effects of the bed bug bites?

1. None ( )
2. Mild ( )
3. Severe ( )

3.12. If severe, how did you manage(medication) them?

3.13. Which pesticide is commonly available and pocket friendly do you often use?

3.14. Was the pesticide effective enough?

1. Yes ( )
2. No ( )

3.15. If NOT, which measures did you take?

3.16. In the household items, where is the highest incidence of the bugs?

1. Furniture ( )
2. Mattresses/bedroom ( )
3. Cracks/Crevices ( )
4. Clothes ( )
5. Others Specify

3.17. Does the infestation affect your self-esteem and social life in general?

1. Yes ( )
2. No ( )

3.17. How do the general community around you perceive bed bug infestations?

1. Weird ( )
2. Normal ( )
3. Any other specify ( )

3.19. After what duration do you notice the bug bites?

1. Immediately ( )
2. 30mins-1hr ( )
3. Any other specify ( )

3.20. At what time of the day are the bugs most active?

1. Morning ( )
2. Midday ( )
3. Night ( )

MODULE 4

**4.1 INFRASTRUCTURE (*all distances in walking minutes*)**

4.1.1 Give the estimated distance to the following community infrastructure and services centers from your residences

| **Infrastructure** | **Distance (Minutes)** |
| --- | --- |
| Village market |  |
| Nearest source of insecticides and pesticides (dealers) |  |
| Nearest neighboring household |  |
| Nearest health center |  |

Time finished interview (24 HR) …………………….

**Thank you very much for your time and participation (Please remember to thank the farmer genuinely)**

**The enumerator to answer section 14 below privately immediately after the interview**

- 1. Did you ask questions properly? [____] 0=No 1=Yes
  2. Overall, how did the respondent give answers to the questions [ __ ]

| 1=willingly | 2=reluctantly | 3=with persuasion | 4=it was hard to get answers |
| --- | --- | --- | --- |

- 1. How often do you think the respondent was telling the truth [ ___ ]?

| 1=rarely | 2=sometimes | 3=most of the times | 4=all the time |
| --- | --- | --- | --- |

**Checked by Supervisor:**

I (supervisor)________________________certify that I have checked the questionnaire to be sure that all the questions have been answered, and that the answers are legible.

Signed: ___________________________________ Date___/___/_____

**Appendix B**

Table S1: Parameters for models’ simulations

| Parameter | Definition | Value |
| --- | --- | --- |
| *Homogeneous houses community* | |  |
| β | Infestation at the rate | 0.4 |
| γ | Infested houses extinction rate. | 0.3 |
| τ | Infested houses treatment rate | 0.2 |
| α | Protection lost rate | 0.5 |
| *Heterogeneous houses community* | |  |
| β_g_ | Infestation rate in good houses | 0.2 |
| γ_g_ | Infested good houses extinction rate. | 0.4 |
| τ_g_ | Infested good houses treatment rate | 0.05 |
| α_g_ | Protection lost rate in good houses | 0.5 |
| β_b_ | Infestation rate in bad houses | 0.5 |
| γ_b_ | Infested bad houses extinction rate. | 0.2 |
| τ_b_ | Infested bad houses treatment rate | 0.1 |
| α_b_ | Protection lost rate in bad houses | 0.5 |

**Table S2 List of environmental variables**

| Variables | Abbreviation | Unites |
| --- | --- | --- |
| Isothermality (BIO2/BIO7) (* 100) | bio3 | ℃ |
| Temperature Seasonality (standard deviation *100) | bio4 | ℃ |
| Max Temperature of Warmest Month | bio5 | - |
| Min Temperature of Coldest Month | bio6 | ℃ |
| Temperature Annual Range (BIO5-BIO6) | bio7 | ℃ |
| Mean Temperature of Wettest Quarter | bio8 | ℃ |
| Mean Temperature of Driest Quarter | bio9 | ℃ |
| Mean Temperature of Warmest Quarter | bio10 | ℃ |
| Mean Temperature of Coldest Quarter | bio11 | ℃ |
| Precipitation of Wettest Month | bio13 | Mm |
| Precipitation of Driest Month | bio14 | Mm |
| Precipitation of Wettest Quarter | bio16 | Mm |
| Precipitation of Driest Quarter | bio17 | Mm |
| Precipitation of Warmest Quarter | bio18 | Mm |
| Precipitation of Coldest Quarter | bio19 | Mm |

| **Table S3.** Study of respondents with bite effects, community perception, self-esteem impact, disinfestation frequency, infested areas, duration of encounter, post-disinfestation disappearance duration, control strategy and cases of relocation due to infestations. | | | |
| --- | --- | --- | --- |
| **Bedbug knowledge, infestation and control practices variables** | | **n** | **(%)** |
| **Bite effect** | |  |  |
|  | None | 21 | 2.3 |
|  | Mild | 620 | 68.9 |
|  | Severe | 259 | 28.8 |
| **Community perception** | |  |  |
|  | Weird | 719 | 79.9 |
|  | Normal | 180 | 20 |
| **Disinfestation frequency** | |  |  |
|  | Once | 92 | 10.2 |
|  | > once | 808 | 89.8 |
| **Most infested areas** | |  |  |
|  | Bedroom/Mattresses | 306 | 34 |
|  | Furniture | 252 | 28 |
|  | Cracks/crevices | 216 | 24 |
|  | Clothes | 126 | 14 |
| **Duration of encounter** |  |  |  |
|  | 1 - 5 Years | 689 | 76.6 |
|  | 6 - 10 Years | 174 | 19.3 |
|  | 11 - 15 Years | 22 | 2.4 |
|  | 16 - 20 Years | 14 | 1.6 |
|  | > 20 Years | 1 | 0.1 |
| **Post-disinfestation disappearance duration** |  |  |  |
|  | 1 Month | 464 | 51.6 |
|  | 2 Months | 218 | 24.2 |
|  | 3 Months | 92 | 10.2 |
|  | ≥ 4 Months | 125 | 13.9 |
| **No. of insecticides used** |  |  |  |
|  | None | 77 | 8.6 |
|  | One | 333 | 37 |
|  | Two | 367 | 40.8 |
|  | Three | 102 | 11.3 |
|  | Four | 19 | 2.1 |
|  | > Four | 1 | 0.1 |
| **Control strategy** |  |  |  |
|  | Chemical and cultural^1^ | 714 | 79.3 |
|  | Cultural only^2^ | 93 | 10.3 |
|  | Chemical only^3^ | 83 | 9.2 |
|  | Botanicals^4^ | 10 | 1.1 |
| **Have you ever relocated because of an infestation?** |  |  |  |
|  | Yes | 456 | 50.7 |
|  | No | 444 | 49.3 |
| **Does the infestation affect your self-esteem and social life in general?** |  |  |  |
|  | Yes | 871 | 96.8 |
|  | No | 29 | 3.2 |

**Table S4.** Recommended and received medication

| **Recommended medication** | **n** | **Percentage (%)** |
| --- | --- | --- |
| Anti-allergies | 91 | 10.1 |
| Anti-inflammatory drugs | 149 | 16.6 |
| Painkillers | 101 | 11.2 |
| Robb ointment | 78 | 8.7 |
| Dettol | 60 | 6.7 |
| Others | 421 | 46.8 |

| **Table S5.** Pesticides used by respondents for bedbug control | | | | |
| --- | --- | --- | --- | --- |
| **Pesticide trade name** | **Active ingredient** | **WHO class^a^** | **Frequency** | **Respondents’ application (%)** |
| Sevin dududust | Carbaryl 7.5% | II | 230 | 25.57 |
| Green leaf | Fipronil | II | 142 | 15.73 |
| Marathion II | Imidacloprid 21.4% | II | 108 | 12 |
| Diazinone | O,O-diethyl- O-(2-isopropyl-6-methyl-4-pyrimidinyl) phosphorothioate. |  | 70 | 7.8 |
| Dortor Doom | 6% pyrethrin | II | 68 | 7.56 |
| Gladiator | Chlorpyrifos 480g/l | II | 64 | 7.08 |
| kungunil | Imidacloprid 200g/l | II | 39 | 4.32 |
| lava | Dichlorvos 1000g/L | Ib | 39 | 4.20 |
| Flavor | Unspecified | N/A | 26 | 2.88 |
| Dursban | Chlorpyrifos | II | 25 | 2.76 |
| Ectomin | cypermethrin high-cis 100g/l | II | 19 | 2.16 |
| Montem Doom | Allethrin (2.09 g/kg​) and Resmethrin (0.39 g/kg) | II | 15 | 1.68 |
| Duduthrin | Lambda-cyhalothrin 17.5 g/lt. | II | 13 | 1.44 |
| Ortho | Bifenthrin 0.05%, Zeta-Cypermethrin 0.01% | II | 9 | 0.96 |
| Bedlam | Acetamiprid 200g/l | II | 8 | 0.84 |
| Dudu kwisha | Unspecified | N/A | 8 | 0.84 |
| Nozzle | Unspecified | N/A | 5 | 0.60 |
| Loyalty | Imidacloprid 700 g/kg | II | 4 | 0.48 |
| Promax | Propoxur 20% W/V | II | 3 | 0.36 |
| Ricatrix | Unspecified | N/A | 3 | 0.36 |
| Triatix | Amitraz | II | 3 | 0.36 |
| ^a^WHO classification: Ia = Extremely hazardous; Ib = Highly hazardous; II = Moderately hazardous; III = slightly hazardous; U = Unlikely to present acute hazard in normal use; FM = Fumigant, not classified; O = Obsolete as a pesticide, not classified. | | | | |


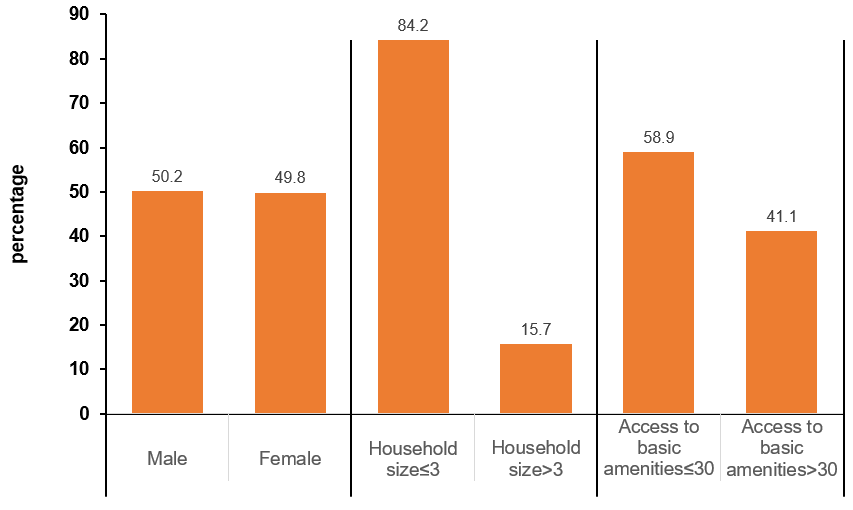


**Figure S1:** The socio-demographic profile of the respondents
